# Determination of Volatile Compounds of *Mentha piperita* and *Lavandula multifida* and Investigation of Their Antibacterial, Antioxidant, and Antidiabetic Properties

**DOI:** 10.1155/2022/9306251

**Published:** 2022-06-14

**Authors:** Samiah Hamad Al-Mijalli, Eman R. ELsharkawy, Emad M. Abdallah, Munerah Hamed, Nasreddine El Omari, Shafi Mahmud, Mohammed Merae Alshahrani, Hanae Naceiri Mrabti, Abdelhakim Bouyahya

**Affiliations:** ^1^Department of Biology, College of Sciences, Princess Nourah Bint Abdulrahman University, P.O. Box 84428, Riyadh 11671, Saudi Arabia; ^2^Chemistry Department, Northern Border University Saudi Arabia, Arar, Saudi Arabia; ^3^Department of Science Laboratories, College of Science and Arts, Qassim University, Ar Rass, Saudi Arabia; ^4^Department of Pathology, Faculty of Medicine, Umm Al-Qura University, Makkah, Saudi Arabia; ^5^Laboratory of Histology, Embryology, and Cytogenetic, Faculty of Medicine and Pharmacy, Mohammed V University in Rabat, Rabat, Morocco; ^6^Division of Genome Sciences and Cancer, The John Curtin School of Medical Research and The Shine-Dalgarno Centre for RNA Innovation, The Australian National University, Canberra, ACT 2601, Australia; ^7^Department of Clinical Laboratory Sciences, Faculty of Applied Medical Sciences, Najran University, 1988, Najran 61441, Saudi Arabia; ^8^Laboratory of Pharmacology and Toxicology, Bio Pharmaceutical and Toxicological Analysis Research Team, Faculty of Medicine and Pharmacy, Mohammed V University in Rabat, BP 6203, Rabat, Morocco; ^9^Laboratory of Human Pathologies Biology, Department of Biology, Faculty of Sciences, and Genomic Center of Human Pathologies, Faculty of Medicine and Pharmacy, Mohammed V University in Rabat, Rabat, Morocco

## Abstract

*Mentha piperita* and *Lavandula multifida* are widely used in Moroccan traditional medicine for the treatment of diabetes and infectious diseases. The aims of this work were the determination of the chemical composition of *Mentha piperita* (MPEO) and *Lavandula multifida* (LMEO) essential oils and the evaluation of their antibacterial, antioxidant, and antidiabetic activities. The chemical composition was determined by GC-MS analysis. The antibacterial effects were evaluated against several bacterial strains using disc diffusion, MIC, and MBC methods. The antioxidant activity was evaluated *in vitro* using DPPH, H_2_O_2_, and xanthine oxidase, and the antidiabetic activity was estimated by the inhibitory effects of *α*-amylase, *α*-glucosidase, and lipase activities. GC-MS results showed that the main compounds of MPEO were menthone (29.24%), levomenthol (38.73%), and eucalyptol (6.75%). However, eucalyptol (28.11%), 2-bornanone (11.57%), endo-borneol (7.82%), and linalyl acetate (5.22%) are the major compounds of LMEO. The results exhibited important inhibitory effects against some bacterial strains with MIC = MBC = 0.39 mg/mL for MPEO against *Staphylococcus aureus* ATCC. However, LMEO exhibited remarkable antioxidant and antidiabetic activities compared to MPEO. Indeed, LMEO inhibited DPPH, H_2_O_2_, and xanthine oxidase with concentrations of 15.23, 21.52, and 8.89 *µ*g/mL, respectively. Moreover, LMEO exhibited *α*-amylase and *α*-glucosidase at IC_50_ = 85.34 and IC_50_ = 59.36 *µ*g/mL, respectively. The findings showed that both MPEO and LMEO exhibit promising biological properties. However, the application of these species or their main bioactive compounds requires further investigation.

## 1. Introduction

Despite the significant advancements in modern medicine, traditional medicine continues to attract the interest of people all over the globe. Up to 80% of the world's population relies on traditional medicine as their main health care supplier [1, 2]. Unfortunately, modern medicine has been unable to stop the rapid spread of infectious illnesses. According to the World Health Organization, infectious illnesses are still the second biggest cause of mortality worldwide [3]. The demand for novel antimicrobial agents and antibiotics, on the other hand, is higher than ever [4–6]. Pathogenic infections are continually evolving in terms of genetic characteristics, complicating the development of antibiotics and vaccines against infectious diseases. This genetic adaptability allows a wide range of pathogenic organisms to change or mutate into more lethal forms that humans cannot fight off [7, 8]; besides, antibiotic development is declining [9]. The United States Food and Drug Administration's (FDA) approval of novel antimicrobial drugs has been reduced by more than 50% [10].

On the other hand, complex intrinsic diseases and oxidative stress-related diseases are emerging currently and are inducing major problems in human health. Indeed, oxidative stress produces free radicals at cellular and molecular levels, which promotes several diseases, including diabetes and cancer. Therefore, the screening of antioxidant natural products is a very promising strategy to prevent and treat these complex diseases [11–13].

Medicinal plants contain a variety of phytochemical compounds that exert distinct physiological effects on the human body and may serve as a source for novel antimicrobial agents. These bioactive compounds include phenolic compounds, flavonoids, alkaloids, diterpenes, triterpenes, naphthoquinones, and sesquiterpene lactones [14, 15].

*Lavandula latifolia* Medicus (spike lavender), a member of the Lamiaceae family, is a fragrant shrub native to the Mediterranean area and cultivated worldwide for its oil, which is known as lavender oil [16]. The essential oil of lavender is widely utilized in massage, aromatherapy, fragrance, and cosmetics. Many animal and human studies support its use as a sedative, anxiolytic, and mood modulator. Lavender oil has antibacterial properties and may be used to treat germs, fungus, and insects. The essential oil of lavender has spasmolytic properties in smooth muscle, which supports its traditional usage as a natural digestive aid [17, 18].

*Mentha piperita* L. (peppermint) is a popular plant whose oils are extensively utilized for a variety of applications. It is used in cosmeceuticals, personal hygiene products, meals, and pharmaceutical items for both flavoring and aroma qualities. It is also used to treat irritation and inflammation. It is also used in aromatherapy, mouthwashes, bath preparations, toothpaste, chewing gum, and topical preparations [19, 20]. Rosmarinic acid and various flavonoids, including eriocitrin, hesperidin, and luteolin, are phenolic elements of the leaves. Menthol and menthone are the two major volatile components of the essential oil of *Mentha piperita* [21]. The purpose of this study was to examine some biological properties (antibacterial, antioxidant, and antidiabetic activities) of two commonly used Moroccan medicinal herbs, *Mentha piperita* and *Lavandula multifida*.

## 2. Materials and Methods

### 2.1. Plant Material and Essential Oil Extraction

Aerial parts of two plants, belonging to the *Lamiaceae* family, were collected from the El Gharb region (Morocco). *Mentha piperita and Lavandula multifida* were collected in April (2021) and dried in the dark. EOs were extracted using the hydrodistillation method and then stored at 4°C until experimental use.

### 2.2. Determination of Chemical Compounds by GC-MS

The chemical components of *Mentha piperita* (MPEO) and *Lavandula multifida* (LMEO) essential oils were determined by using gas-chromatography/mass-spectrometry (GC/MS) analysis conditions as described in our latterly published study [22].

### 2.3. Antibacterial Activity

#### 2.3.1. Microorganisms

Three Gram-positive bacteria, *Staphylococcus aureus* ATCC 29213, *Listeria monocytogenes* ATCC 13932, and *Bacillus subtilis* ATCC 6633, and three Gram-negative bacteria, *Escherichia coli* ATCC 25922, *Pseudomonas aeruginosa* 27853, and *Salmonella Typhimurium* ATCC 700408, were used in this study. Microorganisms were cultured by inoculating a loopful from the frozen stock (−20°C) in Mueller–Hinton Agar (Biokar, Beauvais, France) and incubating at 37°C for 24 hours.

#### 2.3.2. Disc-Diffusion Test

The disc diffusion test was used to assess the antibacterial potential of essential oils, with minor modifications to the procedure published previously [22]. To enhance diffusion in the culture medium, the examined essential oils were combined with dimethyl sulfoxide (DMSO) at a concentration of 5%. Simultaneously, a 0.5 McFarland (10^8^ CFU/mL) bacterial solution representing each examined species was produced in sterile saline water (0.9% NaCl) and inoculated on Mueller–Hinton Agar (Biokar, Beauvais, France) plates by swabbing. Then, 10 *µ*L of each essential oil was put onto sterile paper discs with a diameter of 6 mm, with a disc containing 10 *µ*L of DMSO at a concentration of 5% serving as a negative control and 30 *µ*g of chloramphenicol serving as a reference test. After that, all of the plates were incubated for 24 hours at 37°C. The inhibition diameter was measured in millimeters (disk included) after incubation and given as the mean ± standard deviation of three repetitions.

#### 2.3.3. Determination of MIC and MBC

Microbroth dilution on 96-well microplates was used to determine the minimum inhibitory concentration (MIC) of each essential oil following the procedure published by Eloff [23] with minor modifications. In brief, in each microplate row, decreasing amounts of each essential oil were prepared in DMSO using the serial twofold dilution procedure. The microplates were then incubated at 37°C for 24 hours with 20 *μ*L of bacterial suspensions adjusted to 0.5 McFarland and 160 *μ*L of Mueller–Hinton broth (MHB, Biokar, Beauvais, France). After that, the bacterial growth was examined by incubating for 30 minutes at 37°C with 40 *μ*L of 2, 3, 5-triphenyltetrazolium chloride (TTC) (Sigma-Aldrich, Switzerland) at a concentration of 0.2 *µ*g/mL. The TTC uses a red dye to stain the bacteria, indicating which wells have bacterial growth. The MIC was determined using microplate wells with the lowest concentration of essential oils and no evident bacterial growth. The minimal bactericide concentration (MBC) test [24] was determined by subculturing 10 *μ*L from a microplate well that did not show bacterial growth on Mueller–Hinton Agar (Biokar, Beauvais, France), and then incubating the plates at 37°C for 24 hours. The MBC was defined as the lowest concentration that did not cause any growth in the medium. The reference test in this investigation was chloramphenicol (Sigma-Aldrich).

### 2.4. *In Vitro* Antioxidant Activity

The antioxidant activity of the two tested EOs was evaluated using the following three commonly used in vitro complementary assays: DPPH, H_2_O_2_, and xanthine oxidase (XO) methods, and following the same procedures as described previously by the research group [25–27]. Each assay was carried out in triplicate, and allopurinol and ascorbic acid were used as positive controls. In each assay, the concentrations of the essential oils (EOs) that provided 50% inhibition (IC_50_) were determined, and their values were presented in *µ*g/mL.

### 2.5. *In Vitro* Antidiabetic Activity

The *in vitro* antidiabetic effects of *Mentha piperita and Lavandula multifida* EOs were determined by measuring their capacity to inhibit *α*-amylase and *α*-glucosidase enzymatic activity, following the same methods as we previously described [28, 29] and the IC_50_ (*µ*g/mL) values were determined.

## 3. Results and Discussion

### 3.1. Chemical Composition

**Figure 1:**
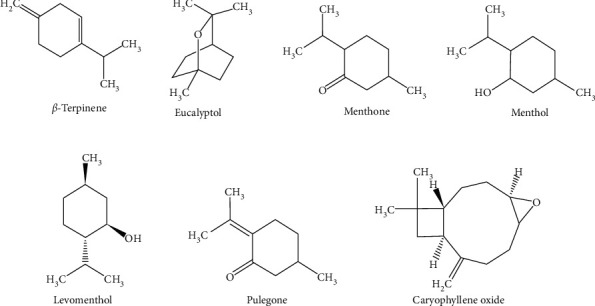
Main compounds of MPEO.

**Figure 2:**
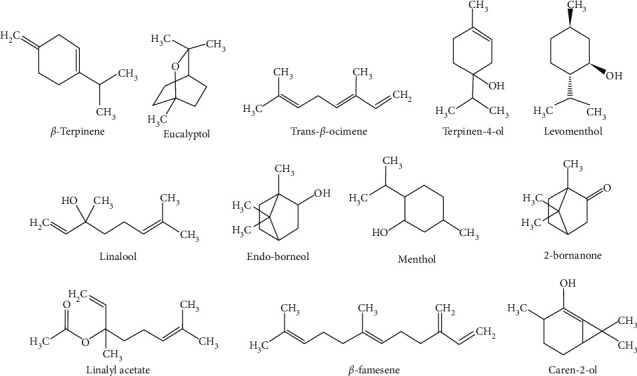
Main compounds of LMEO.

As shown in Table 1, the principal compounds detected in *M. piperita* were menthone (29.24%), levomenthol (38.73%), caryophyllene oxide (3.42%), eucalyptol (6.75%), menthol (2.71%), and germacrene D (0.14%) (Figure 1). In comparison, the major chemical constituents for *L. multifida*. were eucalyptol (28.11%), trans-*β*-ocimene (4.93%), 3-carene (1.86%), *β*-terpinene (2.98%), trans-*β*-ocimene (1.34%), terpinen-4-ol (3.0%), 2-bornanone (11.57%), endo-borneol (7.82%), linalyl acetate (5.22%), linalool (2.89%), and *β*-famesene (4.91%) (Figure 2).

The chemical composition of MPEO contains a high concentration of monoterpene and sesquiterpene, menthone (29.24%), and menthol (38.73%) as the major compounds, which is consistent with the previous study on the harvested Morocco plant, where the chemical composition of EO analyzed by CPG/MS revealed menthol (46.32%), menthone (7.42%), and 1,8-cinole (6.06%) as the main components [30].

The chemical composition of MPEO contains a high concentration of monoterpene and sesquiterpene, with menthone (29.24%) and menthol (38.73%) as the major compounds, which is consistent with previous research on the harvested Moroccan plant, where the chemical composition of EO analyzed by CPG/MS revealed menthol (46.32%), menthone (7.42%), and 1,8-cinole (6.06%) as the main component.

The EO of *L. Multifida* is characterized by the presence of eucalyptol (28.11%), trans-*β*-ocimene (4.93%), 3-carene (1.86%), *β*-terpinene (2.98%), trans-*β*-ocimene (1.34%), terpinen-4-ol (3.0%), 2-bornanone (11.57%), endo-borneol (7.82%), linalyl acetate (5.22%), linalool (2.89%), and *β*-famesene (4.91%). However, the main constituents of the Tunisian L. *multifida* EO were linalool (50.05%), camphene, linalyl acetate, *α*-thujene, bornyl acetate, *β*-caryophyllene, and terpinolene [31], The Algerian plant showed different compositions, carvacrol (65.1%) and *β*-bisabolene (24.7%) [32]. The variability in this chemical composition is due to geographical origin, plant age, extraction, and method of analysis [33, 34].

### 3.2. Antibacterial Activity

**Figure 3:**
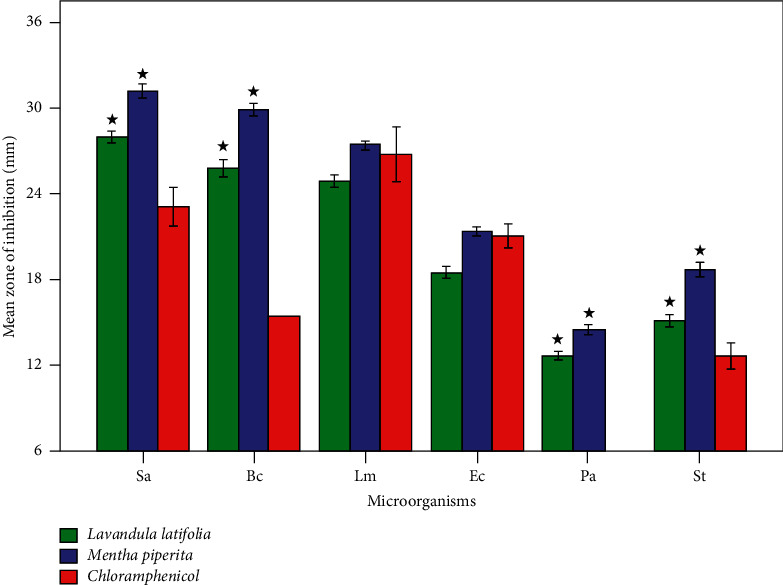
The antibacterial activities of *Lavandula latifolia* and *Mentha piperita* essential oils using disc-diffusion test. Sa: *Staphylococcus aureus* ATCC 29213, Bc: *Bacillus subtilis* ATCC 6633, Lm: *Listeria monocytogenes* ATCC 13932, Ec: *Escherichia coli* ATCC 25922, Pa: *Pseudomonas aeruginosa* 27853, and St: *Salmonella typhimurium* ATCC 700408. ^*∗*^Significant at *P* ≤ 0.05 compared to the chloramphenicol.

Plants have traditionally been the major source of treatments for a broad range of diseases, and one of the most important scientific concerns is the development of novel molecules with antibacterial activity [35]. Our antibacterial testing demonstrated that *Lavandula latifolia* and *Mentha piperita* essential oils are potential sources of antibacterial agents against Gram-positive and Gram-negative bacteria, respectively. The disc diffusion technique was used to determine the antibacterial potential of the essential oils of *Lavandula latifolia* and *Mentha piperita*. The results are presented in Figure 3. The findings indicated that essential oils from the two plants had substantial antibacterial activity against all bacteria tested when compared to the conventional antibiotic (chloramphenicol). *M. piperita* was shown to have stronger antibacterial properties than L. *latifolia*. Gram-positive bacteria were more sensitive than Gram-negative bacteria. In terms of Gram-positive bacteria, *Staphylococcus aureus* had the largest mean zone of inhibition (31.1 ± 0.2 and 27.9 ± 0.2 mm), followed by *Bacillus subtilis* (29.8 ± 0.2 and 25.8 ± 0.3 mm) and *Listeria monocytogenes* (27.4 ± 0.1 and 24.9 ± 0.2 mm) for *M. piperita* and *L. latifolia*, respectively. Regarding the Gram-positive bacteria, *Escherichia coli* had the largest mean zone (21.3 ± 0.1, 18.4 ± 0.2 mm) followed by *Pseudomonas aeruginosa* (14.4 ± 0.1, 12.6 ± 0.1 mm) and *Salmonella Typhimurium* (18.6 ± 0.2, 15.0 ± 0.2 mm) for *M. piperita* and *L. latifolia*, respectively.

The minimum inhibitory concentrations (MIC) and minimum bactericidal concentrations (MBC) values of the tested essential oils are shown in (Table 2). MIC is the lowest concentration of an antibacterial agent that prohibits the observable growth of a microbe while MBC is the lowest concentration of an antibacterial agent necessary to kill a bacterium. Therefore, in this instance as well, *M. piperita* surpasses *L. latifolia* EO as a bactericidal agent. The Gram-positive bacteria tested had the lowest MIC and MBC, with comparable values (MIC = 0.78, MBC = 0.39). Gram-negative, on the other hand, indicated varying degrees of MIC and MBC ratios. *E. coli* had the lowest MIC and MBC values, followed by *S. typhimurium* and *P. aeruginosa*, respectively (Table 2).

Previous research validates our results. Mentha piperita was reported to possess significant broad-spectrum antibacterial properties [36–39]. On the other hand, data on *Lavandula latifolia*'s antibacterial activity is scarce. However, it was reported that *Lavandula latifolia* essential oil had strong antibacterial activity against a variety of bacterial pathogens and that this activity was significantly enhanced when coupled with camphor oil [40]. Notably, the Lavandula genus has over 39 species and approximately 400 recognized cultivars, several of which have antimicrobial activity against bacteria and fungi [41]. For example, the essential oils of *Lavandula heterophylla* demonstrated significant antibacterial action against a variety of bacteria, including *Staphylococcus aureus*, MRSA, *Streptococcus pyogenes*, *Proteus vulgaris*, *Citrobacter freundii*, *Escherichia coli*, and *Pseudomonas aeruginosa* [42]. The *Lavandula stoechas* cultivars that came from Thailand have remarkable antibacterial activity against *Klebsiella pneumoniae* and *Salmonella typhimurium* [43]. Moreover, numerous *in vitro* and *in vivo* investigations indicate that EOs from different medicinal plants have remarkable antimicrobial effects against a broad spectrum of pathogens [44]. Essential oils with antibacterial activity may have benefits over antibiotics in that their antibacterial properties may have beneficial effects on the digestive organs, such as preventing the spread of potentially harmful microbes without influencing the normal flora or regulating dysbiosis [45].

Our study also showed that the MIC values of *Lavandula latifolia* and *Mentha piperita* essential oils were less than the referenced antibiotic (chloramphenicol), meaning a great inhibitory effect. This breakthrough finding has the potential to be exploited in the food or pharmaceutical industries, as well as in synergistic formulations with other natural products, to develop novel antibacterial agents to combat antibiotic-resistant organisms. As a conclusion, more future pharmacological investigations into these two plants are recommended.

### 3.3. Antioxidant Activity

The antioxidant activity of essential oils (EOs) of MP and LM was evaluated by DPPH, H_2_O_2_, and xanthine oxidase. The percentages of inhibition of DPPH, H_2_O_2_, and xanthine oxidase were mathematically modelled to calculate IC_50_s (Table 3). LM essential oil (LMEO) showed significant antioxidant activity compared to MP essential oil (MPEO) in the three tests. Indeed, LMEO inhibited DPPH, H_2_O_2_, and xanthine oxidase with concentrations of 15.23, 21.52, and 8.89 *µ*g/mL, respectively, while MPEO significantly reduced DPPH (IC_50_ = 53.19 *µ*g/mL), H_2_O_2_ (IC_50_ = 34.81 *µ*g/mL), and xanthine oxidase (IC_50_ = 19.74 *µ*g/mL). The results obtained with the DPPH and H_2_O_2_ tests are considered promising compared to those recorded by ascorbic acid, used as a control. Furthermore, the inhibition of xanthine oxidase induced by allopurinol, used as a standard, was important (IC_50_ = 1.24 *µ*g/mL).

Previous investigations have shown that both plants exert varying antioxidant activities [46–50]. The results of these studies do not completely corroborate those obtained in our investigation. This is attributed, of course, to the origin of the plant material, the plant part used, the harvest period, the phenological stage, and the extraction and storage conditions. Indeed, as indicated in the phytochemical section, a remarkable variation of the bioactive compounds is observed in the different studies carried out on both plants, which justifies the fluctuations of the results. Additionally, some studies have highlighted correlations between major compounds and antioxidant activity. In our study, the antioxidant effects recorded are certainly attributed to the presence of certain majority compounds in the aerial parts of MP such as levomenthol (38.73%) and menthone (29.24%), and of LM such as eucalyptol (28.11%) and 2-bornanone (11.57%). Indeed, pharmacological studies have already reported the antioxidant activities of these compounds [51–53].

The antioxidant mechanisms of volatile compounds are not fully elucidated compared to polyphenols and flavonoids, which exert, according to several recent investigations, an antioxidant activity through their ability to share single electrons, which justifies the activity of essential oils not having this property against DPPH and H_2_O_2_.

On the other hand, the remarkable inhibitory activity against xanthine oxidase reinforces this suggestion and shows that volatile compounds inhibit this enzyme via a competitive or noncompetitive interaction. Indeed, terpenoids are considered as inhibitors of enzymatic biomarkers involved in human pathologies, including xanthine oxidase [54, 55], which has, in addition to its oxidizing activity, considerable inflammatory activity. In addition, its inhibition will constitute a preventive pathway against stress and chronic inflammation.

### 3.4. Antidiabetic Activity

*α*-Amylase and *α*-glucosidase are two carbohydrate-hydrolyzing enzymes, and the inhibition of their activity will be a promising therapeutic approach in reducing blood glucose levels and, consequently, in the management of type 2 diabetes mellitus (T2DM). Indeed, LMEO showed a higher antidiabetic effect than MPEO with IC_50_ values of 85.34 and 59.36 *µ*g/mL against *α*-amylase and *α*-glucosidase, respectively.  Table 4 shows that the activity of these two plants was very different from that of acarbose, which we used as a standard, indicating remarkable antidiabetic activity.

In contrast, the inhibition of lipid degradation limits their absorption. In fact, lipid concentrations indirectly regulate glucose concentrations; a decrease in lipids implies a significant breakdown of sugars, which lowers blood glucose. Dietary lipids in the intestine are broken down by lipases before being absorbed. Indeed, the specific inhibition of this enzyme prevents the degradation and, therefore, the decrease in lipid concentration. In this sense, lipases constitute a major therapeutic target for the pharmaceutical industry to fight against the majority of metabolic disorders, including T2DM.

In our study, MP and LM essential oils were tested against lipase and the results showed inhibition with IC_50_ values of 71.36 and 30.94 *µ*g/mL, respectively. These results remain promising when compared with orlistat (IC_50_ = 11.25 *µ*g/mL), the standard reference. Other peppermint species have also shown significant anti-hyperglycemic effects [56–58].

In terms of Lavandula species, a recent study evaluated the inhibitory potential of *Lavandula angustifolia* leaf EO against the enzyme *α*-glucosidase, finding an inhibition of the activity of this digestive enzyme with an IC_50_ of 609.44 *μ*g/mL compared to acarbose (IC_50_ of 526.5 *μ*g/mL [59]. Concerning the *in vivo* tests, the administration of increasing doses of *Lavandula stoechas* hydroalcoholic extracts (50, 100, and 150 mg/kg·b.w.) to alloxan-induced diabetic mice significantly reduced blood glucose concentration in these animals in a dose-dependent manner [60]. In another more recent study, the antihyperglycemic activity of *Lavandula pedunculata* aqueous extract was investigated *in vivo* using an oral glucose tolerance test (OGTT) and an *in vitro* test targeting the two enzymes, pancreatic *α*-amylase and intestinal *α*-glucosidase. As a result, this extract improved rat oral glucose tolerance while inhibiting pancreatic *α*-amylase (IC_50_ = 0.44 ± 0.05 *μ*g/mL) and intestinal *α*-glucosidase (IC_50_ = 131 ± 20 *μ*g/mL) [61]. The promising results of these two species are likely due to their major bioactive molecules, which have been scientifically shown to have the antidiabetic potential [62]. These molecules were found and determined in the phytochemical section.

## 4. Conclusion and Perspectives

Here, we report the chemical composition and biological activities of MPEO and LMEO. Both species showed a variability in volatile compounds with a predominance of phenolic bioactive compounds. The findings showed that MPEO exhibits remarkable antibacterial effects compared to LMEO, which suggests the presence of antibacterial compounds in MPEO. LMEO and MPEO showed important *in vitro* antioxidant effects, with more efficiency for LMEO. Moreover, LMEO exhibited remarkable *in vitro* antidiabetic effects compared to MPEO. Results suggest the use of MPEO and LMEO to develop bioactive molecules with antibacterial, antidiabetic, and antioxidant properties. Future perspectives should be addressed to confirm and validate the biological and pharmacological properties of LMEO and MPEO main compounds. Indeed, studies concerning the mechanisms of action against pathogenic bacteria and others related to antidiabetic effects and oxidative stress-related diseases should be investigated importantly to determine the pharmacodynamic action of each compound. Moreover, toxicological investigations using different doses at different times should also be tested to confirm the safety of both EOs and their main bioactive compounds.

## Figures and Tables

**Table 1:** Chemical composition of MPEO and LMEO.

Number	Compounds	RT	MPEO	LMEO
1	*α*-Pinene	2.07	0.41	0.30
2	Camphene	2.2	0.03	0.74
3	*β*-Terpinene	2.72	0.95	2.98
4	2,3-Dehydro-1,8-cineole	3.01	—	0.16
5	*α*-Phellandrene	3.12	0.06	0.34
6	3-Carene	3.4	—	1.86
7	Eucalyptol	3.918	6.75	28.11
8	Trans-*β*-ocimene	4.31	—	4.93
9	Terpinen-4-ol	4.69	0.27	3.0
10	Linalool	6.08	0.23	2.89
11	2-Bornanone	6.71	—	11.57
12	Menthone	7.15	29.24	—
13	Endo-borneol	7.2	—	7.82
14	Menthol	7.35	2.71	1.90
15	Terpinen-4-ol	7.46	—	7.65
16	Levomenthol	8.22	38.73	3.26
17	Caren-2-ol	8.89	0.15	3.24
18	Pulegone	9.09	1.07	—
119	*D*-Carvone	9.21	0.11	0.15
20	Linalyl acetate	9.77	—	5.22
21	Bornyl acetate	10.15	—	0.07
22	Lavandulyl acetate	10.67	—	1.35
23	Elemene	11.52	0.11	—
24	*α*-Copaene	12.07	0.20	—
25	*β*-Bourbonene	12.89	0.38	—
26	Geranyl acetate	13.50	—	0.35
27	Caryophyllene	13.71	—	1.45
28	*β*-Copaene	13.98	0.20	—
29	Germacrene *D*	14.28	0.24	0.15
30	*α*-Bergamotene	14.32	—	0.18
31	*β*-Famesene	14.91	0.16	4.91
32	*γ*-Muurolene	14.98	0.39	
33	Lavandulyl butyrate	15.3	—	1.02
34	*β*-Bisabolene	15.6	—	0.14
35	*α*-Curcumene	15.99	0.86	
36	Caryophyllene oxide	16.06	3.42	0.22
37	(+)-*β*-Himachalene oxide	16.9	0.06	—
38	Caryophylla-4(12),8(13)-dien-	17.20	0.13	—
39	Cadinol	17.31	—	0.21
40	Alloaromadendrene	17.35	0.17	—
41	Aromadendrene oxide	17.44	0.14	—
42	*α*-Bisabolol	17.91	—	0.53
43	*p*-Menth-8-en-3-ol, acetate	18.03	0.07	—
44	Culmorin	18.69	0. 41	—
	Total		96.04	97.7
	Others		3.96	2.3

**Table 2:** MIC and MBC values of MPEO and LMEO.

Bacteria	LMEO (mg/mL)		MPEO (mg/mL)		Chloramphenicol (*μ*g/mL)
	MIC	MBC	MIC	MBC	MIC
*Staphylococcus aureus* ATCC 29213	0.78	0.78	0.39	0.39	4.0
*L. monocytogenes* ATCC 13932	0.78	0.78	0.39	0.78	2.0
*Bacillus subtilis ATCC* 6633	0.78	0.78	0.39	0.39	32.0
*Escherichia coli ATCC* 25922	1.56	3.12	1.56	1.56	4.0
*Salmonella typhimurium* ATCC 700408	6.25	12.5	3.12	6.25	64.0
*Pseudomonas aeruginosa* ATCC 27853	25	25	12.5	25	>64.0

**Table 3:** Antioxidant activity using DPPH, H_2_O_2_, and xanthine oxidase (XO) methods of MPEO and LMEO.

EOs	DPPH IC_50_ (*µ*g/mL)	H_2_O_2_ IC_50_ (*µ*g/mL)	Xanthine oxidase IC_50_ (*µ*g/mL)
*Mentha piperita*	53.19 ± 1.12^c^	34.81 ± 0.01^c^	19.74 ± 0.08^c^
*Lavandula multifida*	15.23 ± 0.05^b^	21.52 ± 0.07^b^	8.89 ± 0.05^b^
Ascorbic acid	10.73 ± 0.82^a^	4.43 ± 0.02^a^	—
Allopurinol	—	—	1.24 ± 0.02^a^

^*∗*^Different superscript letters in the same column indicate significant difference (*p* < 0.05).

**Table 4:** The IC_50_ values of MPEO and LMEO on *α*-amylase, *α*-glucosidase, and lipase inhibition.

EOs	IC_50_ (*µ*g/mL)		
	*α*-Amylase	*α*-Glucosidase	Lipase
*Mentha piperita*	98.12 ± 0.05^c^	103.48 ± 0.06^c^	71.36 ± 0.02^c^
*Lavandula multifida*	85.34 ± 0.02^b^	59.36 ± 0.03^b^	30.94 ± 0.08^b^
Acarbose	71.54 ± 0.06^a^	39.47 ± 0.02^a^	—
Orlistat	—	—	11.25 ± 0.07^a^

^*∗*^Different superscript letters in the same column indicate significant difference (*p* < 0.05).

## Data Availability

The data used to support the findings of this study are included within the article.
